# Prevalence of Eye Diseases and Causes of Visual Impairment in School-Aged Children in Western China

**DOI:** 10.2188/jea.JE20110063

**Published:** 2012-01-05

**Authors:** Lian-Hong Pi, Lin Chen, Qin Liu, Ning Ke, Jing Fang, Shu Zhang, Jun Xiao, Wei-Jiang Ye, Yan Xiong, Hui Shi, Xi-Yuan Zhou, Zheng-Qin Yin

**Affiliations:** 1 Department of Ophthalmology, Children’s Hospital, Chongqing Medical University, Chongqing, People’s Republic of China; 2 Department of Ophthalmology, the Second Affiliated Hospital, Chongqing Medical University, Chongqing, People’s Republic of China; 3 Southwest Hospital, Southwest Eye Hospital, Third Military Medical University, Chongqing, People’s Republic of China

**Keywords:** school-aged child, refractive error, visual impairment

## Abstract

**Background:**

The present study investigated the prevalence of refractive error, visual impairment, and eye diseases in school-aged children in western China.

**Methods:**

The survey was done in a representative county (Yongchuan District, Chongqing Municipality) of western China. Cluster random sampling was used to select children aged 6 to 15 years. We conducted door-to-door surveys and eye examinations including optometry, stereoscopic vision test, eye position and eye movement, slit lamp examination of the anterior segment, retinoscopy, and fundus examination after cycloplegia with 1% cyclopentolate.

**Results:**

Among 3469 children, data were available for 3079 (88.76%). The prevalences of eye diseases were, in descending order, refractive error (20.69%; 637/3079), conjunctivitis (11.76%; 362/3079), amblyopia (1.88%; 58/3079), color vision defect (0.52%; 16/3079), keratitis (0.36%; 11/3079), strabismus (0.29%; 9/3079), cataract (0.23%; 7/3079), pathologic myopia (0.19%; 6/3079), and ocular trauma (0.13%; 4/3079). The prevalence of corneal leucoma, corneal staphyloma, optic neuropathy, macular degeneration, and myelinated nerve fibers was 0.03% (1/3079) for each. The prevalence of visual impairment was 7.70% (237/3079), and the major causes of visual impairment were uncorrected refractive error (86.08%; 204/237), amblyopia (9.70%; 23/237), pathologic myopia (1.27%; 3/237), congenital cataract (0.42%; 1/237), and others (2.11%; 5/237).

**Conclusions:**

Among school-aged children in a less developed area of western China, refractive error was the most prevalent eye disorder, and uncorrected refractive error was the main cause of visual impairment.

## INTRODUCTION

Vision is critical for daily activities, and visual impairment is one of the most serious disabilities. Visual impairment at birth or during childhood can affect learning, communication, employment, health, and quality of life, and the effects are often life-long.^1^

Although the proportion of infants and school-aged children with visual impairment is less than 5%, children with visual impairment account for 20% of individuals with visual impairment worldwide, after adjustment for disability-adjusted life years (DALYs).^2^ The control of visual impairment and blindness in children is a priority of the World Health Organization’s VISION 2020 program.^3^

Uncorrected refractive error is one of the most common causes of visual impairment in children. Increasing evidence indicates that uncorrected refractive error is a main cause of avoidable blindness in many regions, including Chile,^4^ Africa,^5^ and Malaysia.^6^ In China, refractive error and visual impairment have been surveyed during the past decade, and the findings confirm that refractive error is a predominant cause of visual impairment.^7^^,^^8^ However, further studies are required to explain these findings, due to differences in methodology, population characteristics, living environment, socioeconomic development, availability of primary health care, and ethnicity.

Surveys of visual impairment in Chinese school-aged children are mainly performed in schools and clinics.^9^^,^^10^ Although several population-based surveys of refractive error and other eye diseases have been carried out in developed areas in China, eg, Beijing^11^ and Guangzhou,^7^ those findings cannot be generalized to all of China, especially its less developed areas. To reduce the prevalence of visual impairment and blindness, it is imperative to acquire data on the prevalence of refractive error, visual impairment, and eye diseases among school-aged children of western China.

Western China is a region with a vast territory (6 800 000 km^2^) that accounts for 71% of the land mass of China. This region consists of 11 provinces and 1 municipality but is sparsely populated (360 million people; 28% of the overall Chinese population).^12^ As compared with other regions, western China is less developed. Thus, eye diseases and visual impairment are not carefully monitored, due to the low socioeconomic status and living standards in this region. To date, no epidemiologic study has investigated the prevalence of these diseases. In addition, the causes of visual impairment in children are closely related to socioeconomic status. In the past 3 decades, the Chinese economy has developed rapidly, and it is therefore imperative to investigate the evolution of the causes of visual impairment in children, which will allow better coordination of the prevention and treatment of eye diseases with socioeconomic development.

To determine the prevalence of refractive error, visual impairment, and eye diseases in school-aged children in western China, we conducted a population-based survey of a representative area (the Yongchuang District of Chongqing Municipality) of western China. The population structure of this district is relatively stable, and its socioeconomic status is intermediate for western China. We hope that our findings will provide new data to aid in the prevention and treatment of refractive error and visual impairment among school-aged children in China.

## METHODS

### Sample selection

The survey was conducted in the Yongchuang District of Chongqing Municipality. Chongqing City is an economic and cultural hub of western China. According to the China Fifth National Population Census (2000), the municipality of Chongqing has a population of 30.51 million.^13^ Yongchuang District is one of 40 administrative districts of Chongqing Municipality, has a relatively stable population structure, and a socioeconomic status that is intermediate for western China, which makes it representative of that area. Moreover, an epidemiologic study of glaucoma and cataract in adults that was conducted in this area provided a basis for the present survey.

Neighborhood committees in city and village committees in the countryside were defined as clusters, which was followed by cluster sampling. The total number of children enrolled was 3469, which was higher than previously calculated (3005).^14^ The sample size was estimated as n ≈ Z^2^(ρ)(1 − ρ)/B^2^, where ρ is the rate and B is the tolerable error. Z was calculated according to the 95% confidence interval (CI). The rate was 20% of the prevalence of refractive error in our pilot study, the permissible error was 1.5%, and the detection rate was about 90%.^15^

### Field survey

Families with eligible children were selected using the Population Census Data from 2000. Written informed consent was obtained from the parents of all children before the study. This study conformed to the Declaration of Helsinki and was approved by the World Health Organization Research Ethics Review Committee, the local Ethics Committee, and the Education and Health Bureaus of Yongchuang District.

Subject screening was performed from 8 August 2006 to 5 September 2006. Demographic information was obtained from village/neighborhood committees and validated door-to-door. A notice of the time and place where the children would receive examinations was sent to the parents or legal guardians.

A pilot study was carried out from 6 September to 7 October 2006 and enrolled 324 children aged 6 to 15 years. In that pilot study, the examiner became familiar with the equipment and workflow, which provided a basis for this subsequent survey. The prevalence of refractive error in the pilot study was 20%, which was used to estimate the present sample size. The survey was performed from 8 October 2006 to 1 January 2007.

### Eye examination

The eye examinations were performed by a team of 3 nurses, 2 ophthalmologists, and 1 optometrist. The vision test, stereoscopic vision test, test of the anterior segment and eye movement, eye drop instillation, retinoscopy, and fundus examination were done in different rooms. Details of the examination procedures have been previously reported.^14^

### Inclusion and exclusion criteria

*Enrollment of participants:* demographic information was collected from the local neighborhood/rural committees. Children aged 6 to 15 years were recruited. Children not registered locally were nevertheless recruited if they had lived in the local area for longer than 6 months. Those who had not lived in the local area for longer than 6 months were excluded, even if they were registered locally. Those temporarily leaving the local area, and sick children, were also enrolled in the present study. Children meeting the following criteria were recruited: age 6 to 15 years at examination; informed consent form signed by parents or legal guardians; and no history of cardiovascular or nervous system diseases, including congenital heart diseases, hypoxic ischemic encephalopathy, and cognitive impairment. Children were excluded if they had a history of cardiovascular or nervous system diseases, including congenital heart diseases, hypoxic ischemic encephalopathy, and cognitive impairment; if informed consent was not obtained before the study or their parents/legal guardians refused the examinations; if they could not fix their gaze during testing; or if they did not cooperate with the examination or could not recognize the items on the vision test card. Children meeting any exclusion criterion were excluded from the study.

### Statistical analysis

Spherical equivalent (SE) was defined as the sum of spherical power and half-cylinder power in diopters. The spherical equivalent in all age groups is expressed as mean ± SD of both eyes and median diopters because the distributions for the left and right eyes were similar (Pearson correlation coefficient = 0.90). However, extreme diopter values were rare for the left eye; thus, it better represents the overall trend. Therefore, we only report data for the left eye.

In addition to grouping participants by age bracket, the children were classified into 3 age groups: 6 to 8 years (early elementary school), 9 to 12 years (late elementary school), and 13 to 15 years (junior high school). This allowed us to investigate similarities among children of different ages. One-way analysis of variance (ANOVA) and the least significant difference (LSD) post-hoc test were used to compare SEs among age groups. The Kolmogorov-Smirnov test was used to confirm normal distribution of SEs among multiple age groups. The software package SPSS version 13.0 was used for the statistical analysis. The prevalences of hyperopia, myopia, and astigmatism were analyzed by using the chi-square test. The prevalences of eye diseases and visual impairment were calculated, and the cause of visual impairment was analyzed. Prevalence of visual impairment was compared by using the chi-square test. In all tests, a *P* value of less than 0.05 was considered to indicate statistical significance.

### Related definitions and criteria

*Spherical equivalent:* algebraic sum of spherical power and half-cylinder power, ie, sphere + cylinder/2.

*Hyperopia:* SE refraction of +2.00 D or more^16^^,^^17^ in 1 or both eyes.

*Myopia:* SE refraction of −0.50 D or more^16^^,^^17^ in 1 or both eyes.

*Astigmatism:* absolute cylindrical value of 1.00 D of cylinder or more in 1 or both eyes.

*Prevalence:* total number of cases in the population divided by the number of individuals in the population.

*Daily life visual acuity (presenting visual acuity):* distance visual acuity.^7^^,^^18^ For children who never wore glasses, uncorrected visual acuity was recorded. For those who occasionally wore glasses, daily life visual acuity was defined as uncorrected visual acuity. For those wearing glasses, daily life visual acuity was defined as corrected visual acuity.

*Visual impairment:* a daily life visual acuity in the better eye of 20/40 or worse.^7^^,^^18^ The better eye was defined as the eye with superior visual acuity on the vision examination.

*Amblyopia:* a best corrected visual acuity in 1 or both eyes that was lower than the normal visual acuity for children of the same age, in the absence of organic changes. Usually, the best corrected visual acuity was 20/25 or worse in children with amblyopia.^19^

*Pathologic myopia:* a condition with myopia and myopia-specific or nonspecific pathologic changes.^20^ The criteria for the diagnosis of myopia were: a family history of myopia (immediate family member with high-degree myopia); myopia development in early childhood; degree of myopia −4.25 D or worse in children aged 6 to 10 years or −6.00 D or worse in children aged 12 to 15 years; 4) rapid development of myopia, 5) fundus examination showing changes in the fundus, including slanted optic disk, lacquer crack formation, and Fuch’s spot.^21^

### Quality control

The pilot study was conducted to familiarize the staff with the workflow, confirm the performance of the equipment, and coordinate the activities of the personnel. Each examination was done by the same examiner. This minimized systematic error, which can occur when 1 examiner does several examinations or several examiners share 1 examination. The accuracy and integrity of the demographic and clinical data were double-checked before inputting them into the database. The data were input twice by 2 examiners to confirm correct input.

## RESULTS

### Study population

We enrolled 28 clusters, which included 3611 families. There were 3469 children aged 6 to 15 years in 2552 of these 3611 families: 1 child in each of 1713 families and 2 or more children in each of 839 families. Among the 3469 children, 390 were excluded from the study for the following reasons: refusal to participate, eye discomfort (burning sensation, photophobia, irritation, etc) that interfered with the examination, other pathologic conditions (congenital cerebral diseases, cardiovascular diseases, etc), noncooperation, and dysgnosia. Ultimately, 3079 children completed the entire study, including 1615 boys (52.45%) and 1464 girls (47.55%); the ratio of males to females was 1.1:1.0. The mean age of the children was 10.41 ± 2.73 years, and the detection rate was 88.76%. The detection rate in girls (90.88%) was higher than in boys (86.92%). Table 1 shows the detection rates by sex and age. In addition, the 324 children in the pilot study were also included in the 3079 children.

**Table 1.** Age and sex distribution of the selected and examined populations

Age	NO. (%) of All			NO. (%) of Boys			NO. (%) of Girls		
		
	Selected	Examined	%Exam	Selected	Examined	%Exam	Selected	Examined	%Exam
6	300 (8.65)	239 (7.79)	79.67	177 (9.53)	139 (8.63)	78.53	123 (7.64)	100 (6.85)	81.30
7	362 (10.44)	315 (10.23)	87.02	195 (10.50)	170 (10.53)	87.18	167 (10.37)	145 (9.90)	86.83
8	369 (10.64)	339 (11.04)	91.87	170 (9.15)	156 (9.68)	91.76	199 (12.35)	183 (12.54)	91.96
9	378 (10.90)	351 (11.40)	92.86	196 (10.55)	181 (11.21)	92.35	182 (11.30)	170 (11.65)	93.41
10	373 (10.75)	342 (11.11)	91.69	166 (8.93)	154 (9.56)	92.77	207 (12.85)	188 (12.84)	90.82
11	349 (10.06)	321 (10.43)	91.98	200 (10.76)	181 (11.21)	90.50	149 (9.25)	140 (9.56)	93.96
12	358 (10.32)	306 (9.94)	85.47	197 (10.60)	167 (10.37)	84.77	161 (10.00)	139 (9.49)	86.34
13	325 (9.37)	287 (9.32)	88.31	181 (9.74)	157 (9.72)	86.74	144 (8.94)	130 (8.88)	90.28
14	379 (10.93)	354 (11.53)	93.40	207 (11.14)	187 (11.61)	90.34	172 (10.68)	167 (11.45)	97.09
15	276 (7.96)	225 (7.33)	81.52	169 (9.10)	123 (7.64)	72.78	107 (6.64)	102 (6.99)	95.33
All	3469 (100.0)	3079 (100.0)	88.76	1858 (100.0)	1615 (100.0)	86.92	1611 (100.0)	1464 (100.0)	90.88

### Distribution of refractive status

Mydriasis was induced by a cycloplegic (1% cyclopentolate) and refractive status was measured. The mean SE of left eyes was 0.47 ± 1.20 D. Table 2 shows the SE values and refractive status of left eyes. The mean SE decreased from 1.36 to −0.14 D from age 6 years to 15 years, but the decrease in SE was not consistent among children of different ages. With increasing mean age, median SE progressively declined. Median SE was 1.25 D in children aged 6 years and 0.25 D in those aged 15 years. The box plot in the Figure shows the SE distribution in left eyes by age.

**Figure.**
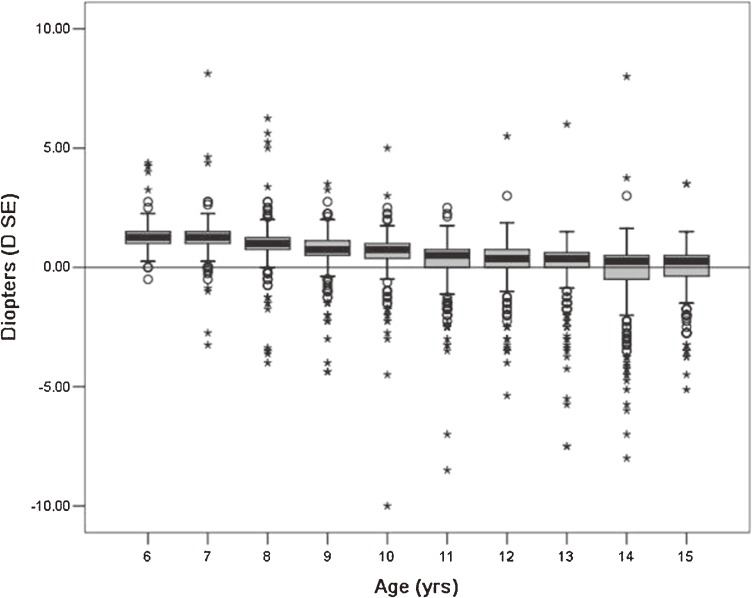
Distribution of spherical equivalent refractive error in left eyes by age, as measured with cycloplegic retinoscopy. Boxes indicate the 25th to 75th percentiles of the age-specific distribution, ie, the interquartile range, and the bars inside the boxes represent the median. The whiskers extend to the lower and upper extremes, defined as the 25th percentile minus 1.5 times the interquartile range and the 75th percentile plus 1.5 times the interquartile range.

**Table 2.** Spherical equivalent refractive error (in diopters) in left eyes

Age (yrs)	Mean^a^	Median	SD	Range	Kolmogorov-Smirnov test		Kurtosis	Skewness
					z-statistic	*P*-value		
Total	0.47	0.75	1.20	−10.00 to 8.13	11.116	<0.001	10.68	−1.80
6	1.36^A^	1.25	0.58	−0.50 to 4.38	3.011	<0.001	9.29	1.92
7	1.22^B^	1.25	0.77	−3.25 to 8.13	3.808	<0.001	27.48	1.52
8	0.94^C^	1.00	0.95	−4.00 to 6.25	3.898	<0.001	12.91	−0.43
9	0.66^D^	0.75	0.87	−4.38 to 3.25	3.785	<0.001	10.06	−2.34
10	0.56^D^	0.75	1.04	−10.00 to 5.00	4.391	<0.001	34.37	−3.84
11	0.21^E^	0.50	1.11	−8.50 to 2.50	4.299	<0.001	17.58	−3.26
12	0.13^EF^	0.37	1.06	−5.38 to 5.50	3.793	<0.001	6.46	−1.43
13	−0.00^F^	0.37	1.30	−7.50 to 6.00	4.524	<0.001	11.15	−2.32
14	−0.23^G^	0.25	1.52	−8.00 to 8.00	4.843	<0.001	6.40	−1.36
15	−0.14^G^	0.25	1.21	−5.13 to 3.50	3.641	<0.001	2.90	−1.36

^a^Means with different letters (A, B, C, D, E, F, G) were significantly different (*P* < 0.05, ANOVA, LSD).

### Prevalence of eye diseases

A total of 1128 children had eye diseases (prevalence, 36.64%). The major eye diseases were refractive error (637, 20.69%), conjunctivitis (362, 11.76%), and amblyopia (58, 1.88%). Table 3 shows the distribution of eye diseases among the 3079 school-aged children.

**Table 3.** Prevalence of eye diseases in children

Eye diseases	Prevalence *n* (%)
Refractive error	637 (20.69)
Hyperopia	100 (3.25)
Myopia	422 (13.71)
Astigmatism	115 (3.73)
Conjunctivitis	362 (11.76)
Amblyopia	58 (1.88)
Ametropic	33 (1.07)
Anisometropic	22 (0.71)
Strabismic	2 (0.06)
Perception	1 (0.03)
Color vision defects	16 (0.52)
Keratitis	11 (0.36)
Strabismus	9 (0.29)
Exotropia	5 (0.16)
Esotropia	3 (0.10)
Vertical	1 (0.03)
Cataract	7 (0.23)
Congenital	3 (0.10)
Traumatic	4 (0.13)
Pathologic myopia	6 (0.19)
Ocular trauma	4 (0.13)
Corneal leucoma	1 (0.03)
Corneal staphyloma	1 (0.03)
Optic neuropathy	1 (0.03)
Macular diseases	1 (0.03)
Myelinated nerve fibers	1 (0.03)
Unknown causes	10 (0.32)
Total	1128 (36.64)

### Prevalence of visual impairment

The distribution of visual impairment is shown in Table 4. Among 3079 children, the prevalence of visual impairment was 7.70%. There was no marked difference in the prevalence of visual impairment between girls (8.68%) and boys (6.99%) (χ^2^ = 2.83, *P* = 0.09). In addition, the prevalence of visual impairment varied by age. Younger age was associated with lower prevalence: 4.03% in children aged 6 to 8 years, 7.65% in those aged 9 to 12 years, and 11.55% in those aged 13 to 15 years. The difference among groups was significant (χ^2^ = 34.91, *P* < 0.001), and there significant differences between all pairs of groups (*P* < 0.01 for all comparisons).

**Table 4.** Prevalence of visual impairment in school-aged children by age group and sex

	Children with visual impairment	Children without visual impairment	All children	Statistical analysis	
	*n* (%)	*n* (%)	*n*	χ^2^	*P* value^a^
Sex					
Male	112 (6.99)	1504 (93)	1616	2.83	0.09
Female	125 (8.68)	1338 (91.32)	1463		
Age					
6–8 years	36 (4.03)	857 (95.97)	893	34.91	<0.001
9–12 years	101 (7.65)	1219 (92.35)	1320		
13–15 years	100 (11.55)	766 (88.45)	866		
All	237 (7.70)	2842 (92.30)	3079		

^a^*P* values were calculated using the chi-square test.

### Cause of visual impairment

Table 5 shows the causes of visual impairment. The most common cause of visual impairment was uncorrected refractive error, which accounted for 86.08% (204/237) of cases; amblyopia was the second most common cause (9.70%; 23/237). Other causes of visual impairment were uncommon.

**Table 5.** Causes of visual impairment in school-aged children

Cause	Children presenting with visual impairment (1 or both eyes) Number (%)	Percent prevalence in the population (1 or both eyes) %
Uncorrected refractive error	204 (86.08)	6.63
Amblyopia	23 (9.70)	0.75
Myopia disease	3 (1.27)	0.10
Congenital cataract	1 (0.42)	0.03
Unexplained causes^a^	5 (2.11)	0.16
Total	237 (100.00)	7.70

^a^Cause could not be determined due to limitations of the examination and the need for more comprehensive testing.

## DISCUSSION

In the present study, the Yongchuang District of Chongqing Municipality was selected because it is representative of the population, geographic location, and socioeconomic status of western China. Among the 3469 children identified, 3079 completed the entire study. The detection rate was 88.76%, which confirms the quality of the study. The detection rate was high because there are an insufficient number of optometrists and ophthalmologists in this area and few children receive free eye examinations. Thus, the study had a high level of acceptability among children and parents. In addition, this study obtained the support of the government, Health Bureau, and Education Bureau. Furthermore, the sites where examinations were performed were accessible and had convenient transportation, which resulted in the active participation of children and parents.

The analysis of refraction showed negative bias at 8 years: a slight negative bias was noted at 8 years (skewness coefficient = −0.43) and an obvious negative bias was observed at 9 years (skewness coefficient = −2.34). This change may be attributable to an increase in children with myopia. Thus, we speculate that age 8 to 9 years is a key age bracket in the development of myopia. In addition, our results revealed that age was related to the prevalence of hyperopia and myopia, ie, with increasing age, the prevalence of hyperopia decreased but that of myopia increased. Among children younger than 9 years, hyperopia was more prevalent than myopia; however, among children aged 9 years or older, myopia was more prevalent than hyperopia. This change is further evidence that age 8 to 9 years is a critical period in the distribution of refraction. Therefore, greater attention to children in this age group might delay the occurrence and development of myopia.

Our results show that the most common eye disease was refractive error, which accounted for 20.69% of eye diseases. This finding suggests that refractive error is a common cause of eye diseases among school-aged children and should thus be monitored. In our previous study, we noted that learning intensity was strongly related to myopia and that close reading could promote development of myopia.^14^ Conjunctivitis was the second most common cause of eye diseases. Although conjunctivitis does not usually result in blindness, it is the main cause of absence from school.^22^ In addition, conjunctivitis can affect quality of life. The Yongchuang District is a suburban district of Chongqing municipality and hygiene is relatively poor. Some children are unaware of eye health and sometimes rub their eyes with dirty hands, which is the main cause of conjunctivitis.

The prevalence of amblyopia was 1.88% (58/3079), which was lower than in Mexican children aged 12 to 13 years (2.5%)^23^ and Iranian school-aged children (2.32%),^24^ but higher than in Sudanese school-aged children (0.92%)^25^ and Swedish children aged 12 to 13 years (1.1%).^26^ This may be due to the exclusion of children with amblyopia who recovered. In this survey, some parents mentioned that their children had a history of amblyopia that resolved after treatment. Additionally, age at the time of survey and the criteria used to define amblyopia might have affected the results.

The prevalence of strabismus was 0.29%, which was similar to estimates for a Nigerian population aged 4 to 24 years (0.3%)^27^ and a Ghanaian population aged 6 to 22 years (0.2%),^28^ but lower than that in Chinese children aged 5 to 15 years living in a different region (4.9%),^9^ in South African children aged 5 to 15 years,^5^ and in Brazilian children aged 11 to 14 years (1.07%).^29^ Ethnicity, geographic location, age, and other population characteristics might have affected the prevalence of strabismus. In addition, in this study, the degree of strabismus was measured only at a short distance, which is another possible reason for the discrepancy between this and other studies. The prevalence of cataract was relatively low (0.23%) and was similar to that in Jordanian children aged 6 to 14 years (0.2%)^30^ and Tanzanian children aged 7 to 19 years (0.22%),^31^ but lower than that in Nigerian children younger than 15 years (2.9%).^32^ The population studied and participant age might explain differences in the prevalence of cataract. In addition, it is possible that some infants with cataract were abandoned or cared for in welfare homes and orphanages and thus were not included in the present analysis. This might also explain the discrepancy in cataract prevalence between the present study and other studies. Additionally, in recent years, prenatal and postnatal care has been emphasized by the Chinese government and perinatal health care has also been enhanced, which has decreased cataract prevalence. However, this must be confirmed in future studies.

Based on a daily life visual acuity in the better eye of worse than 20/40, the prevalence of visual impairment was 7.70%, which was lower than estimates in Chinese children in a different region (10.3%),^7^ a Malaysian study (10.1%), and a Chilean report (14.7%),^4^ but higher than in a Brazilian report (2.67%),^29^ an African study (1.2%),^5^ and a Turkish report (1.7%).^33^ These findings reminds us that the quality of life of children in western China is a concern. Our results revealed no significant correlation between sex and prevalence of visual impairment. A study by Attebo and Mitchell found that the prevalence of visual impairment was higher in females than in males.^34^ However, Michon and Lau reported that prevalence was higher in males.^35^ Yusup et al^36^ proposed that the sex difference in the prevalence of visual impairment is the result of differences in constitution, population distribution, social status of females, income, and other factors. In the present study, population-based sampling was performed, the sample size was large, and there was gender balance. In addition, there was no sex discrimination; thus, girls and boys had equal educational opportunities.

In this population, uncorrected refractive error was the most common cause of visual impairment, accounting for 86.08% of cases, followed by amblyopia (9.7%). The prevalence of congenital eye diseases was relatively low. The prevalence of refractive error, especially myopia, rose with increasing age, and uncorrected refractive error and amblyopia were the first and second most common causes of visual impairment, respectively. These findings were similar to those of population-based studies.^6^^–^^8^^,^^37^ Therefore, correction of refractive error and treatment of amblyopia should substantially improve the prevalence of visual impairment.

The results of comparisons of prevalence of visual impairment among children of different ages showed that it increased with age, which might be associated with the increase in myopia prevalence with age and the lack of timely correction of refractive error.

### Limitations

In the present study, only retinoscopy was used for refraction examination, which could have resulted in intraobserver error. In addition, we did not record sociodemographic characteristics that might have been associated with refractive error, eg, the education level of parents and family income. In addition, although the area from which the population was selected included a school for the blind and an orphanage, no children from those facilities were enrolled, which might have biased the analysis of the distribution of eye diseases. Additionally, our results showed that 839 families had 2 or more children. Certain eye diseases, and especially myopia, have a strong genetic background. Children in the same family share many lifestyle factors, which could have biased the results of the present study.

## References

[1] Brown MM, Brown GC, Sharma S, Busbee B Quality of life associated with visual loss: a time tradeoff utility analysis comparison with medical health states. Ophthalmology. 2003;110:1076–81 10.1016/S0161-6420(03)00254-9 12799229

[2] Sun BC. Low vision in clinical practice. Beijing: Huaxia Publishing House. 1999;36:138,154–9.

[3] Gilbert C, Foster A Childhood blindness in the context of VISION 2020—the right to sight. Bull World Health Organ. 2001;79:227–32 11285667 PMC2566382

[4] Maul E, Barroso S, Munoz SR, Sperduto RD, Ellwein LB Refractive error study in children: results from La Folrida, Chile. Am J Ophthalmol. 2000;129:445–54 10.1016/S0002-9394(99)00454-7 10764851

[5] Naidoo KS, Raghunandan A, Mashige KP, Govender P, Holden BA, Pokharel GP, Refractive error and visual impairment in African children in South Africa. Invest Ophthalmol Vis Sci. 2003;44:3764–70 10.1167/iovs.03-0283 12939289

[6] Goh PP, Abqariyah Y, Pokharel GP, Ellwein LB Refractive error and visual impairment in school-age children in Gombak District, Malaysia. Ophthalmology. 2005;112:678–85 10.1016/j.ophtha.2004.10.048 15808262

[7] He M, Zeng J, Liu Y, Xu J, Pokharel GP, Ellwein LB Refractive error and visual impairment in urban children in southern China. Invest Ophthalmol Vis Sci. 2004;45:793–9 10.1167/iovs.03-1051 14985292

[8] He M, Huang W, Zheng Y, Huang L, Ellwein LB Refractive error and visual impairment in school children in rural southern China. Ophthalmology. 2007;114:374–82 10.1016/j.ophtha.2006.08.020 17123622

[9] Hornby SJ, Xiao Y, Gilbert CE, Foster A, Wang X, Liang X, Causes of childhood blindness in the People’s Republic of China: results from 1,131 blind school students in 18 provinces. Br J Ophthalmol. 1999;83:929–32 10.1136/bjo.83.8.929 10413695 PMC1723134

[10] He B, Hao Q Clinical analysis of children’s ocular disease. J Norman Bethune Univ Med Sci. 2001;27:661–2

[11] Zhao J, Pan X, Sui R, Munoz SR, Sperduto RD, Ellwein LB Refractive Error Study in Children: results from Shunyi District, China. Am J Ophthalmol. 2000;129:427–35 10.1016/S0002-9394(99)00452-3 10764849

[12] National Bureau of Statistics of China. China statistical yearbook 2003. Beijing: China Statistics Press; 2003. p. 100–2 (in Chinese).

[13] Chongqing Municipal Bureau of Statistics. Chongqing statistical yearbook 2004. Beijing: China Statistics Press; 2004. p. 58–9 (in Chinese).

[14] Pi LH, Chen L, Liu Q, Ke N, Fang J, Zhang S, Refractive status and prevalence of refractive errors in suburban school-age children. Int J Med Sci. 2010;7:342–53 20975844 10.7150/ijms.7.342 PMC2962262

[15] Negrel AD, Maul E, Pokharel GP, Zhao J, Ellwein LB Refractive error study in children: sampling and measurement methods for a multi-country survey. Am J Ophthalmol. 2000;129:421–6 10.1016/S0002-9394(99)00455-9 10764848

[16] Anera RG, Soler M, de la Cruz Cardona J, Salas C, Ortiz C Prevalence of refractive errors in school-age children in Morocco. Clin Experiment Ophthalmol. 2009;37:191–6 10.1111/j.1442-9071.2009.02001.x 19723127

[17] Robaei D, Kifley A, Rose KA, Mitchell P Refractive error and patterns of spectacle use in 12-year-old Australian children. Ophthalmology. 2006;113(9):1567–73 10.1016/j.ophtha.2006.02.066 16857261

[18] Sun BC, Lu Q, Zheng YY Discussion on the new WHO classification of visual impairment. Ophthalmol China. 2005;14

[19] Ge J, Zhao JL, Cui H. Ophthalmology. Beijing: People’s Medical Publishing House; 2005. p. 400–1.

[20] Wang FR, Yin ZG. Myopia, eyes with myopia and pathological myopia. Shanghai: Fudan University Press; 2008. p. 7–9.

[21] Li FM. Chinese Ophthalmology. (Book II) (2nd edition). Beijing: People’s Medical Publishing House; 2005. p. 2418–9.

[22] Onwasigwe EN, Umeh RE, Onwasigwe CN Referral pattern of children to the eye department of the University of Nigera Teaching Hospital, Enugu, Nigeria. Nig J Ophth. 1996;1:5–6

[23] Ohlsson J, Villarreal G, Sjöström A, Cavazos H, Abrahamsson M, Sjöstrand J Visual acuity, amblyopia, and ocular pathology in 12- to 13-year-old children in Northern Mexico. J AAPOS. 2003;7:47–53 10.1016/S1091-8531(02)42011-3 12690370

[24] Yekta A, Fotouhi A, Hashemi H, Dehghani C, Ostadimoghaddam H, Heravian J, The prevalence of anisometropia, amblyopia and strabismus in schoolchildren of Shiraz, Iran. Strabismus. 2010;18:104–10 10.3109/09273972.2010.502957 20843187

[25] Lithander J Prevalence of amblyopia with anisometropia or strabismus among schoolchildren in the Sultanate of Oman. Acta Ophthalmol Scand. 1998;76:658–62 10.1034/j.1600-0420.1998.760604.x 9881546

[26] Ohlsson J, Villarreal G, Sjöström A, Abrahamsson M, Sjöstrand J Visual acuity, residual amblyopia and ocular pathology in a screened population of 12-13-year-old children in Sweden. Acta Ophthalmol Scand. 2001;79:589–95 10.1034/j.1600-0420.2001.790609.x 11782225

[27] Ajaiyeoba AI, Isawumi MA, Adeoye AO, Oluleye TS Prevalence and causes of eye diseases amongst students in south-western Nigeria. Ann Afr Med. 2006;5:197–203

[28] Ntim-Amponsah CT, Ofosu-Amaah S Prevalence of refractive error and other eye diseases in schoolchildren in the Greater Accra region of Ghana. J Pediatr Ophthalmol Strabismus. 2007;44:294–7 17913172 10.3928/01913913-20070901-04

[29] Salomão SR, Cinoto RW, Berezovsky A, Mendieta L, Nakanami CR, Lipener C, Prevalence and causes of visual impairment in low-middle income school children in São Paulo, Brazil. Invest Ophthalmol Vis Sci. 2008;49:4308–13 10.1167/iovs.08-2073 18829856 PMC6031127

[30] Maaita JF, Sunna LF, Al-Madani MV, Horrani SM Eye diseases in children in Southern Jordan. Saudi Med J. 2003;24:154–6 12682678

[31] Wedner SH, Ross DA, Balira R, Kaji L, Foster A Prevalence of eye diseases in primary school children in a rural area of Tanzania. Br J Ophthalmol. 2000;84:1291–7 10.1136/bjo.84.11.1291 11049957 PMC1723290

[32] Ezegwui IR, Onwasigwe EN Pattern of eye diseases in children at Abakaliki, Nigeria. Int J Ophthalmol. 2005;5:1128–30

[33] Unsal A, Ayranci U, Tozun M Prevalence of lower urinary tract symptoms among men in a rural district of western Turkey. Pak J Med Sci. 2010;26:294–9

[34] Attebo K, Mitchell P, Smith W Visual acuity and the causes of visual loss in Australia. The Blue Mountains Eye Study. Ophthalmology. 1996;103:357–64 8600410 10.1016/s0161-6420(96)30684-2

[35] Michon JJ, Lau J, Chan WS, Ellwein LB Prevalence of visual impairment, blindness, and cataract surgery in the Hong Kong elderly. Br J Ophthalmol. 2002;86:133–9 10.1136/bjo.86.2.133 11815334 PMC1771025

[36] Yusup M, Chen XY Epidemiology survey of visual loss. Int J Ophthalmol. 2010;10:304–7

[37] Dandona R, Dandona L, Srinivas M, Sahare P, Narsaiah S, Muñoz SR, Refractive error in children in a rural population in India. Invest Ophthalmol Vis Sci. 2002;43(3):615–22 11867575

